# Special Issue: Honey Bee Research in the US: Current State and Solutions to Beekeeping Problems

**DOI:** 10.3390/insects10010022

**Published:** 2019-01-09

**Authors:** Margarita M. López-Uribe, Michael Simone-Finstrom

**Affiliations:** 1 Department of Entomology, Center for Pollinator Research, Pennsylvania State University, University Park, PA 16802, USA; mml64@psu.edu; 2 USDA Agricultural Research Service, Honey Bee Breeding, Genetics and Physiology Research, Baton Rouge, LA 70820, USA

## 1. Introduction

The European honey bee (*Apis mellifera*) is the most important managed species for agricultural pollination across the world. Despite their importance, managed honey bee colonies are experiencing annual mortality rates that now typically range between 30 to 40% in North America and Europe [1,2]. These high overwintering losses have been linked to a myriad of stressors—including pesticides, diseases and poor nutrition [3]—that weaken colony health and are threatening the sustainability of the beekeeping industry. The phenomenon that was described as Colony Collapse Disorder (CCD) in winter 2006/spring 2007 [4] and ultimately the potential negative economic and ecological impacts that could result from a deficit of honey bees for pollination services and honey production [5,6] have spurred a large body of scientific literature on a variety of applied and basic aspects in apicultural research.

The American Association of Professional Apiculturists (AAPA) is an organization consisting of professors, state apiarists, scientists and students who study and work with honey bees. The goals of this organization are to (1) promote communication within and between industry, academia and the beekeeping community, (2) develop and foster research on fundamental and applied questions to gain a greater understanding of honey bee biology that can assist and improve the beekeeping industry; and (3) create a venue to rapidly share new techniques and current research to advance the field. Due to recent problems with high honey bee colony losses, the number of research groups and scientists dedicated to apicultural research has increased during the past 10 years. However, no North American-based peer-reviewed scientific journal is dedicated to the dissemination of the high-quality studies in basic and applied research that are generated every year in this field.

With this special issue, we aimed to create a space to highlight progress and updates of honey bee research in the United States (US), namely by AAPA member research groups. Specifically, we compile studies that address critical and novel questions to advance our understanding of key aspects related to (1) honey bee colony health, (2) abiotic and biotic stressors—with a special focus on larval and adult responses to pesticides and viruses—and (3) approaches to inform beekeeping management practices. Below, we summarize the key findings of these studies and present an overview of current critical research topic needs.

## 2. Honey Bee Colony Health

Identifying reliable biomarkers to characterize colony health is critical for (1) research focusing on improving honey bee health and (2) the translation of these findings into successful beekeeping. One of the most widely used biomarkers of queen health is brood pattern—defined as the shape left by the egg-laying behavior of the queen. Because the reproductive capacity of the queen is fundamental for colony growth, the homogeneity of capped brood in a colony is often used as an indication of the quality of the queen (e.g., more continuous brood indicates a better queen). Lee and colleagues [7] experimentally investigate the correlation between “good” and “poor” brood patterns and other metrics of honey bee health. Contrary to the expected results, these authors found that queens that exhibited “poor” brood pattern were no different than queen with “good” laying patterns in terms of sperm viability, queen size, and colony diseases. This study will challenge the widespread use of brood patterns as a metric of queen health and raises questions about how colony phenotype influences queen health and reproductivity metrics. Another critical driver of colony health is the quality of the drones that will successfully mate with newly emerged queens. Metz and Tarpy [8] tested for the first time the degree of variation in sperm viability among drones reared in controlled conditions and found that the major source of drone viability is determined by age, not by individuals. The discovery of the role of age in drone sperm viability will impact the timing of artificial insemination events in breeding programs. This study also provides critical information for optimal levels of viable spermatozoa, an important biomarker that could impact queen reproductive quality. In the last paper in this section, Brutscher and colleagues [9] summarize the existing literature on copulation factors linked to queen quality and reproduction. Specifically, they carefully review two aspects of copulatory factors in honey bees: (1) how queen behavior, physiology, and expression profiles change after copulation, and (2) the role of semen and seminal fluids on these post-mating changes in queens. Brutscher et al. also identify gaps of knowledge in the interaction between drone fertility and queen reproductive success. These are significant contributions investigating one of the major issues impacting the beekeeping industry—queen reproductive health—yet it has been understudied. These studies highlight the need for further work related to understanding how queen reproductive output is influenced by interacting abiotic and biotic factors.

## 3. Environmental Stressors of Honey Bee Colonies

The increased toxicity and persistence of agrochemicals in the environment have been identified as one of the most interactive stressors affecting honey bee colonies [10]. Evidence for sublethal effects after pesticide exposure at the individual and colony level has been found for a number of key biological traits such as foraging behavior [11,12,13,14] and reproduction [15,16], but these effects are not always consistently noted [17]. One of the growing concerns of pesticide exposure to honey bees is the potential for synergistic interactions between chemicals that have different modes of action. Ostiguy and colleagues [18] present a four-year study of pesticide residue in pollen and wax samples from trials in six regions in the United States. They report high numbers of different fungicides, herbicides, and insecticides in pollen and wax, and high variability between colonies from different regions. In addition, this study finds strong correlations between the presence of insecticides and fungicides that have different modes of action (e.g., disruption of mitosis and cell division and disruption sterol biosynthesis in membranes) highlighting the potential for synergistic effects between different types of pesticides. Wade and colleagues [19] provide experimental evidence for the synergistic lethal effects that common fungicides and insecticides, used in California almond orchards, have on developing larvae. This study finds empirical evidence for the detrimental synergistic effects of insecticides and fungicides applied in combination. On the other hand, Payne and colleagues [20] investigate the potential for negative impacts of initial pesticide contamination in wax on honey bee colony growth or overwintering survival. They found no evidence linking the presence of pesticides or miticides in wax and reduction in colony growth or overwintering success. These results further highlight the need for future, in depth and larger studies on the role of existing pesticide residue in wax as a potential stressor of managed honey bee colonies.

Three papers in this section shed light on individual physiological, molecular, and behavioral responses to pesticide and pathogen exposure. Cook [21] reports data on chronic oral exposure of adult honey bees to sublethal doses of clothianidin and imidacloprid. His results reveal novel and differential metabolic responses after exposure to these two neonicotinoids. Specifically, he finds that while high doses of clothianidin lower glycogen and lipid content in honey bees, high doses of imidacloprid depress honey bee metabolism. These results shed light on the multifactorial effects that may subtly influence bee health and indicate the importance of looking at multiple levels of biological organization when conducting studies related to sublethal stressors. Zhao and colleagues [22] investigate the expression profiles of newly emerged adults after *Varroa destructor* parasitism and subsequent Deformed wing virus (DWV) infection. Their results indicate that the honey bee immune system mounts a large immune response after *V. destructor* and DWV infections, but this response is only temporary and allows rapid viral replication 2 days after initial infection, a finding highlighting mechanisms underlying the interactions between the honey bee immune gene expression, DWV replication, and enhanced *Varroa* reproductive output [23]. Using behavioral assays, Amiri and colleagues [24] examine how colony infections by Israeli Acute Paralysis Virus (IAPV) change queen interactions with infected workers. Their results suggest that queens may interact less frequently with infected workers, but the relationship was not statistically significant. However, they found evidence of queen IAPV infection via contact with infected workers, even though queens exhibited overall lower levels of the virus than infected workers in the same colony. Investigating mechanisms of IAPV resistance in queens may provide a future avenue of research to identify mechanisms of viral resistance in bees. Additionally, this study highlights how behavioral interactions may limit the spread of disease and the need to further evaluate the influence of nestmate partitioning or avoidance as a social immune response [25,26], particularly with respect to reproductive castes.

## 4. Beekeeping Management Practices

The successful survival of managed honey bee colonies is often affected by the practices that beekeepers use to solve problems in the colony (e.g., pests and diseases) and to achieve production goals (e.g., honey production, pollination services). There are a large number of management options available for beekeepers to use. Underwood and colleagues [27] used a national survey dataset to investigate how (1) the size of the beekeeping operation and (2) the philosophy of beekeepers towards the use of chemical treatments to control pests and diseases are associated with beekeeping management practices. Their results indicate that sets of management practices are associated with both factors clearly differentiating approaches used by commercial beekeepers (large operations) and natural beekeepers (chemical-free operations). A common practice among beekeepers who use organic management practices is to irradiate equipment to destroy the presence pathogens in reused comb. de Guzman and colleagues [28] investigate the long-term impact of gamma irradiation of comb on pollen collection, varroa and viral levels, and colony survival. Surprisingly, their results show no significant benefit of comb irradiation for colony health. Indeed, they detected similar levels of virus in irradiated and non-irradiated comb suggesting that this management practice may only have subtle effects on colony health. This study also demonstrates the interactive effects of using *Varroa*-resistant stocks and the influence that the behavioral, and possibly physiological, traits of these mite-resistant bees ultimately have on viral loads in bees, *Varroa*, and wax.

The last two studies of this special issue present research on the value of nutrition as a potential mechanism that could be managed to mitigate stressors such as pesticide and pathogen exposure. Mogren and colleagues [29] led a controlled experiment using larvae from pollen-deprived and pollen-supplemented colonies to determine how different doses of the neonicotinoid clothianidin impacts their levels of oxidative stress. They find that supplemental feeding may be able to mitigate the high levels of oxidative stress triggered by the ingestion of different levels of clothianidin. Using nectar solutions supplemented with phytochemicals, Bernklau and colleagues [30] investigate the ability of bees to survive after infections of the protozoan *Nosema ceranae* when fed with caffeine, gallic acid, kaempferol, and *p*-coumaric acid. Their results show that caffeine significantly reduces infections at low dosage, while infected individuals fed with caffeine and gallic acid lived on average twice as long as control bees. Studies in this section underscore the complexity of how honey bee biology and beekeeping practices interact and impact colony health, and the need to conduct multifactorial analyses at different organizational levels when possible.

## 5. Concluding Remarks

The collection of empirical and review papers in this special issue show that the current challenges of reducing annual honey bee colony losses are a major driver of the apicultural research in the US. However, these studies have also generated a large body of literature on basic aspects of honey bee biology that need to be understood to guide beekeeping practices that can improve honey bee health. Some of the key topics of critical need for apicultural research include better biomarkers of queen health—as it is a major factor impacting the industry, a better understanding of genetic, physiological and behavioral mechanisms of resistance to viruses, and how management practices can help mitigate biotic and abiotic stressors of honey bees. The studies presented in this special issue make some headway in the advancements on these topics and hopefully will inform some meaningful changes into solutions to the current beekeeping problems in the US and the world.
